# A case report of primary pulmonary meningioma masquerading as lung metastasis in a patient with rectal carcinoma: role of ^18^F-FDG PET/CT

**DOI:** 10.1186/s13019-021-01546-3

**Published:** 2021-05-29

**Authors:** Maoqing Jiang, Ping Chen, Rong Huang, Jingfeng Zhang, Jianjun Zheng

**Affiliations:** 1 Ningbo PET/CT center, Hwa Mei Hospital, University of Chinese Academy of Sciences, Ningbo, China; 2 Ningbo Institute of Life and Health Industry, University of Chinese Academy of Sciences, Ningbo, China; 3 Department of Nephrology, Hwa Mei Hospital, University of Chinese Academy of Sciences, Ningbo, China; 4 Department of Pathology, Hwa Mei Hospital, University of Chinese Academy of Sciences, Ningbo, China

**Keywords:** Primary pulmonary meningioma, ^18^F-fluorodeoxyglucose, PET/CT, Metastasis, Rectal carcinoma

## Abstract

**Background:**

Primary pulmonary meningioma (PPM) is an extremely rare disease, which is often misdiagnosed as lung metastasis. Previous studies indicated that PPM usually showed homogeneous enhancement on enhanced CT and high uptake of ^18^F-fluorodeoxyglucose (^18^F-FDG) on positron emission tomography/CT (PET/CT). In this study, we report a case of PPM with atypical enhanced CT and ^18^F-FDG PET/CT features in a patient with rectal carcinoma.

**Case presentation:**

A 70-year-old male was demonstrated to have rectal carcinoma by biopsy while a solitary pulmonary nodule (SPN) with well-defined edges measuring 13 × 13 × 15 mm was almost simultaneously found in the right lower robe on chest CT scan. Contrast-enhanced CT and PET/CT revealed mild centripetal enhancement of the nodule without accumulation of ^18^F-FDG. A thoracoscopic wedge resection of the right lower lobe was finally performed and histopathologic examinations and PET/CT imaging showed that the nodule was a PPM.

**Conclusion:**

PPM is a rare disease with heterogeneity not only in blood supply but also in glucose metabolism. ^18^F-FDG PET/CT may be an effective method for differentiating benign and malignant SPNs. The diagnosis of PPM depends on pathological and radiological examinations.

## Background

Meningioma is a common intracranial neoplasm with an incidence of approximately 2.46 to 5.04 per 100,000, but only 1–2% is primary ectopic meningioma [1, 2]. Primary pulmonary meningioma (PPM) is the most frequently reported extracranial meningioma albeit it is extremely rare [3–5]. It is often misdiagnosed as lung metastasis or malignant tumor partly due to the false positive on positron emission tomography/computed tomography (PET/CT) scan with ^18^F-fluorodeoxyglucose (^18^F-FDG) [6–8]. However, PPM without uptake of ^18^F-FDG on PET/CT has never been reported before. Moreover, it is really difficult to distinguish PPM from isolated lung cancer or metastasis by clinical and radiographic features [9]. Herein, we report a case of a 70-year-old male with rectal carcinoma and contaminant with a solitary and solid peripheral pulmonary nodular without accumulation of ^18^F-FDG that demonstrated to be PPM by pathological examinations.

## Case presentation

**Fig. 1:**
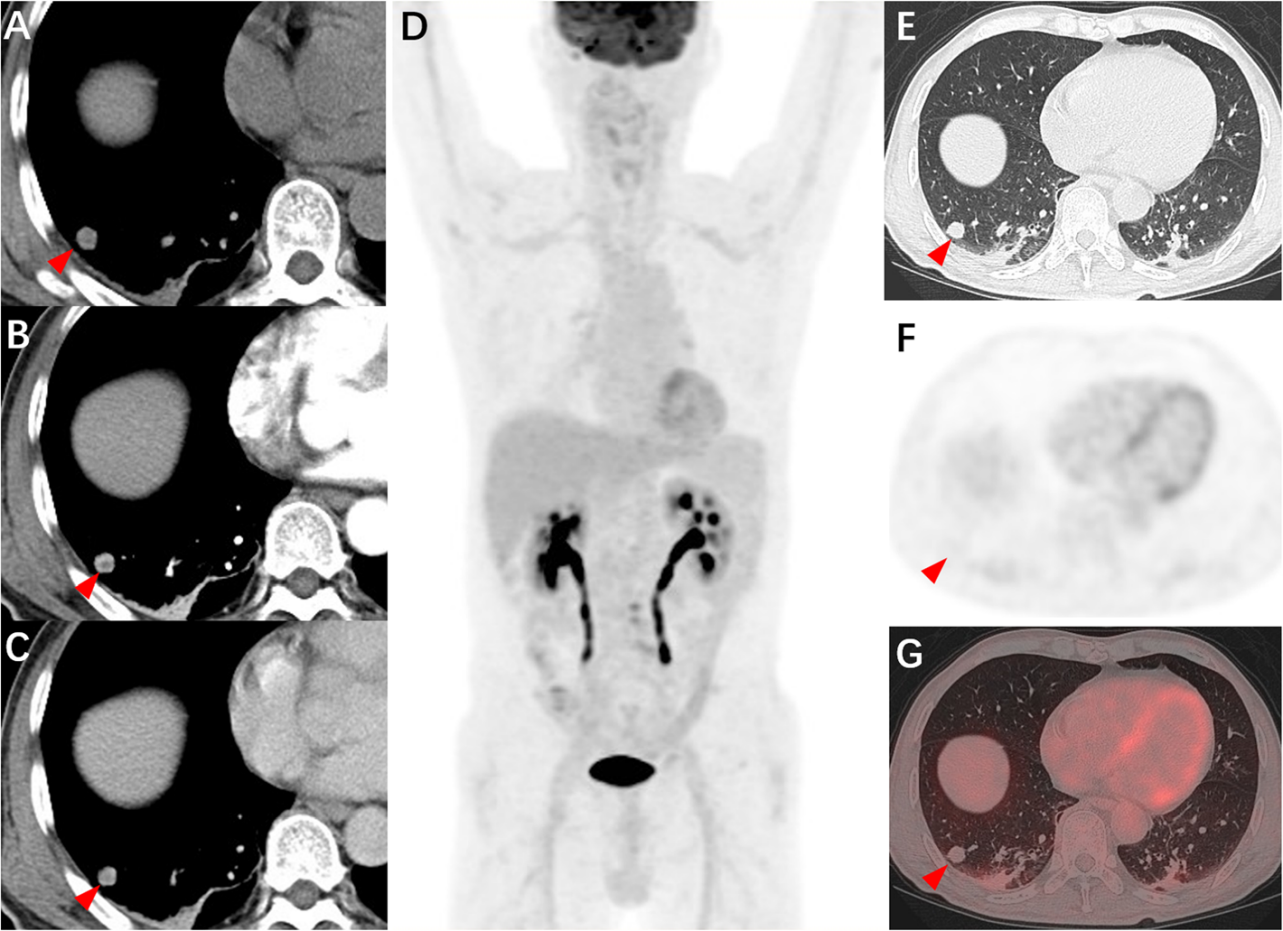
Transverse contrast-enhanced CT scan of the chest (**A**-**C**, red arrowheads) revealing a solitary solid peripheral pulmonary nodular in the right lower lobe with well-defined margins and mild centripetal enhancement. The maximum intensity projection PET Image (**D**) showing normal in the whole body. Transverse chest CT (**E**), corresponding PET (**F**) and fusion PET/CT (**G**) images demonstrating a solitary pulmonary nodular in the right lower lobe with no increased uptake of ^18^F-FDG

A 70-year-old male was diagnosed with rectal carcinoma by biopsy whereas a solitary and solid peripheral pulmonary nodule with well-defined edges measuring 13 × 13 × 15 mm in the right lower robe was incidentally observed on abdominal CT scan (Fig. 1A, arrowhead). No clinical symptoms (e.g., coughing, sputum, hemoptysis and fever) were revealed. Due to the suspicious of metastasis, contrast-enhanced CT scan was performed after radical resection of rectal cancer, which showed mild centripetal enhancement of the nodular (Fig. 1B-C, arrowheads). However, the diagnosis of metastatic tumor could not be completely ruled out. ^18^F-FDG PET/CT was performed for further evaluation. The maximum intensity projection PET image revealed no abnormalities in the whole body (Fig. 1D). Transverse chest CT, corresponding PET and fusion PET/CT (Fig. 1E-G, red arrowheads) images showed a solitary pulmonary nodular (SPN) without accumulation of ^18^F-FDG (maximum standard uptake value, SUVmax 0.6) in the right low lobe, probably a benign tumor. However, a thoracoscopic wedge resection of the right lower lobe was performed 2 months after radical resection of rectal cancer owing to the suspicion of lung metastasis, albeit the possibility was very low.

**Fig. 2:**
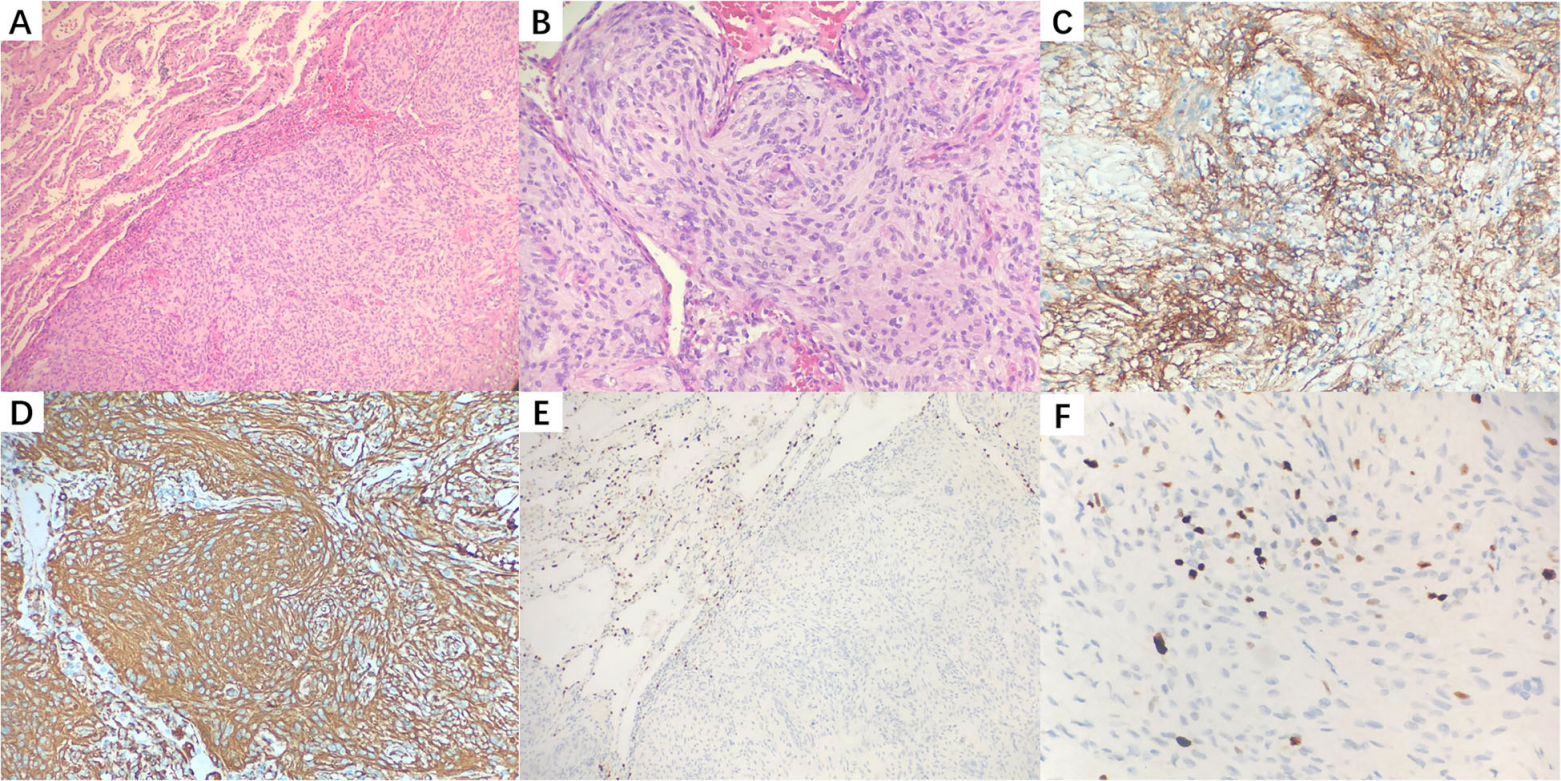
Pathological examination (**A**, Hematoxylin-eosin Stain, Original Magnification 10×) revealing a well-circumscribed nodular with adjacent uninvolved pulmonary parenchyma. Spindle-shaped cells with poorly defined cell borders arranged in whorls (**B**, 20×). On immunohistochemistry, The tumor cells stained positive for EMA (**C**, 20×) and vimentin (**D**, 20×), and negative for TTF1 (**E**, 10×). Ki-67 index was less than 5% (**F**, 20×). These findings are supporting the diagnosis of pulmonary meningioma

Pathological examination (Fig. 2A) revealed a well-circumscribed nodular with adjacent uninvolved pulmonary parenchyma. Spindle-shaped cells with poorly defined cell borders arranged in whorls (Fig. 2B). The tumor cells were immunohistochemically positive for EMA and vimentin, and negative for TTF1 (Fig. 2C-E). Ki-67 index was less than 5% (Fig. 2F). These findings are supporting the diagnosis of pulmonary meningioma (World Health Organization grade I). The final diagnosis of PPM was based on PET/CT images that showed no involvement of central nervous system.

## Discussion

In this study, we report an extremely rare disease, PPM, which shows no significant uptake of ^18^F-FDG on PET/CT scan, a feature that has never been reported before. It is often misdiagnosed as lung metastasis owing to the difficulty of distinguishing the two pathologies on conventional chest CT scan and sometimes due to the high uptake of ^18^F-FDG on PET/CT scan [4, 8, 10]. In our case, it was first regarded as lung metastasis of rectal carcinoma owing to the presentation of solitary pulmonary nodular with well-defined boundary on CT images. On contrast enhanced CT scan, mild centripetal enhancement of the nodular was observed and it was difficult to exclude the diagnosis of metastasis. Kim et al. reported a well-defined pulmonary nodular with intense and homogeneous enhancement that was pathologically confirmed to be PPM [11]. Therefore, the inconsistent enhancement might indicate heterogeneity of blood supply.

The clinical symptoms of PPM vary greatly, mostly asymptomatic [8, 9, 12], sometimes with atypical chest pain [11], but rarely with hemoptysis [13]. In our study, the patient showed asymptomatic and only incidentally observed a solitary pulmonary nodule on abdominal CT scan. From previously published case reports, the clinical symptoms may be related to the location of pulmonary nodules. The distribution area of PPM plays a critical role in clinical symptoms. Most patients present asymptomatic because of the site of PPM that is located in the peripheral pulmonary region, but sometimes it presents with hemoptysis, indicating involvement of venule in the pulmonary parenchyma. It mainly presents as benign tumor, while malignant features, e.g. lymph node, liver or bone metastasis, can also be seen in rare instances [1, 14]. The prognosis of patients with PPM is favorable although it presents as malignancy, possibly due to the slow progression.

^18^F-FDG PET/CT has been demonstrated to be more accurate than conventional CT in evaluating solitary pulmonary nodules (SPNs) [15]. The benign diagnosis of SPN on PET and CT is strongly associated with low uptake of ^18^F-FDG (SUVmax < 1.5–2.0) and well-defined margin [15]. According to this criteria, the SPN in the right lower lobe of our case is probably a benign nodule. However, a thoracoscopic wedge resection of the right lower lobe was performed owing to the suspicion of malignancy, albeit with a very small possibility. The final diagnosis of PPM depends on pathological and radiological examinations showing no involvement of the central nervous system. Interestingly, PPM often showed high uptake of ^18^F-FDG on PET/CT scan in previous case reports [6, 8, 13]. An asymptomatic SPN with increased uptake of ^18^F-FDG in the right upper lobe was reported by Cura et al. that demonstrated to be a pulmonary meningioma [8]. Similarity, Meirelles et al. showed a SPN with a false positive PET scan that proved to be PPM by histopathological examinations [6]. Both were misdiagnosed as primary lung carcinoma. However, no significant uptake of ^18^F-FDG was found in our case, which might indicate the existence of heterogeneity of PPM in glucose metabolism. To our knowledge, it was the first case to report a PPM that showed no uptake of ^18^F-FDG on PET/CT scan in a patient with rectal carcinoma. The absent uptake of ^18^F-FDG might be explained by the low WHO grade of meningioma (grade I) and Ki-67 index [16]. Overall, the diagnosis of pulmonary meningioma still depends on histopathologic examinations, because it’s difficult to make this diagnosis by clinical features and imaging modalities, including ^18^F-FDG PET/CT.

## Conclusion

The present study has reported a rare phenomenon, PPM, in a patient with rectal carcinoma. Our results indicate that the heterogeneity of PPM may exist not only in blood supply but also in glucose metabolism. ^18^F-FDG PET/CT may be an effective method for differentiating benign and malignant SPNs. The diagnosis of PPM depends on pathological and radiological examinations.

## Data Availability

The datasets used and/or analyzed during the current study are available from the corresponding author on reasonable request.

## Abbreviations

PPM: Primary pulmonary meningiomas
SPN: Solitary pulmonary nodule
^18^F-FDG: ^18^F-fluorodeoxyglucose
PET/CT: Positron emission tomography/computed tomography
WHO: World health organization
